# Supplementary material for: Paleocene/Eocene carbon feedbacks triggered by volcanic activity
Source: Nat Commun. 2021 Aug 31;12:5186. doi: 10.1038/s41467-021-25536-0 (PMC8408262; doi:10.1038/s41467-021-25536-0)
Supplement: Supplementary file 1 — Supplementary Information [file 41467_2021_25536_MOESM1_ESM.pdf]

Supplementary Information

Paleocene/Eocene carbon feedbacks triggered by volcanic activity

Sev Kender[1,2*], Kara Bogus[1], Gunver K. Pedersen[3], Karen Dybkjær[3], Tamsin A. Mather[4], Erica Mariani[1], Andy Ridgwell[5], James B. Riding[2], Thomas Wagner[6], Stephen P. Hesselbo[1], Melanie J. Leng[7]

[1]Camborne School of Mines, University of Exeter, Penryn Campus, Penryn, Cornwall TR10 9FE, UK.

[2]British Geological Survey, Keyworth, Nottingham NG12 5GG, UK.

[3]Geological Survey of Denmark and Greenland (GEUS), Øster Voldgade 10, DK-1350 Copenhagen K, Denmark.

[4]Department of Earth Sciences, University of Oxford, Oxford OX1 3AN, UK.

[5]Department of Earth and Planetary Sciences, University of California at Riverside, Riverside, California 92521, USA.

[6]Lyell Centre, Heriot-Watt University, Edinburgh EH14 4AS, UK.

[7]National Environmental Isotope Facility British Geological Survey, Keyworth, Nottingham NG12 5GG, UK.

*Correspondence to: s.kender@exeter.ac.uk

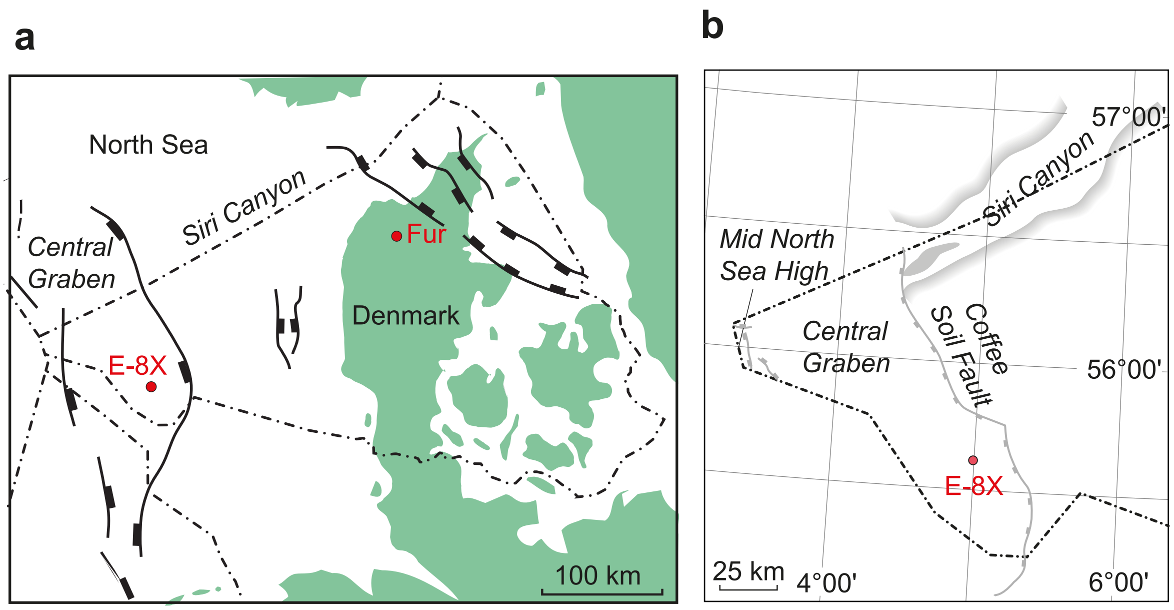

Supplementary Fig. 1. Location maps. a, Location map of present-day Denmark[46] with locations discussed in the text. **b,** Location map of present-day offshore Denmark (Danish Sector of the North Sea Basin is shown in broken line)[46], showing the location of E-8X study core and the position of the Siri paleo-canyon. Main bounding faults shown as black lines.

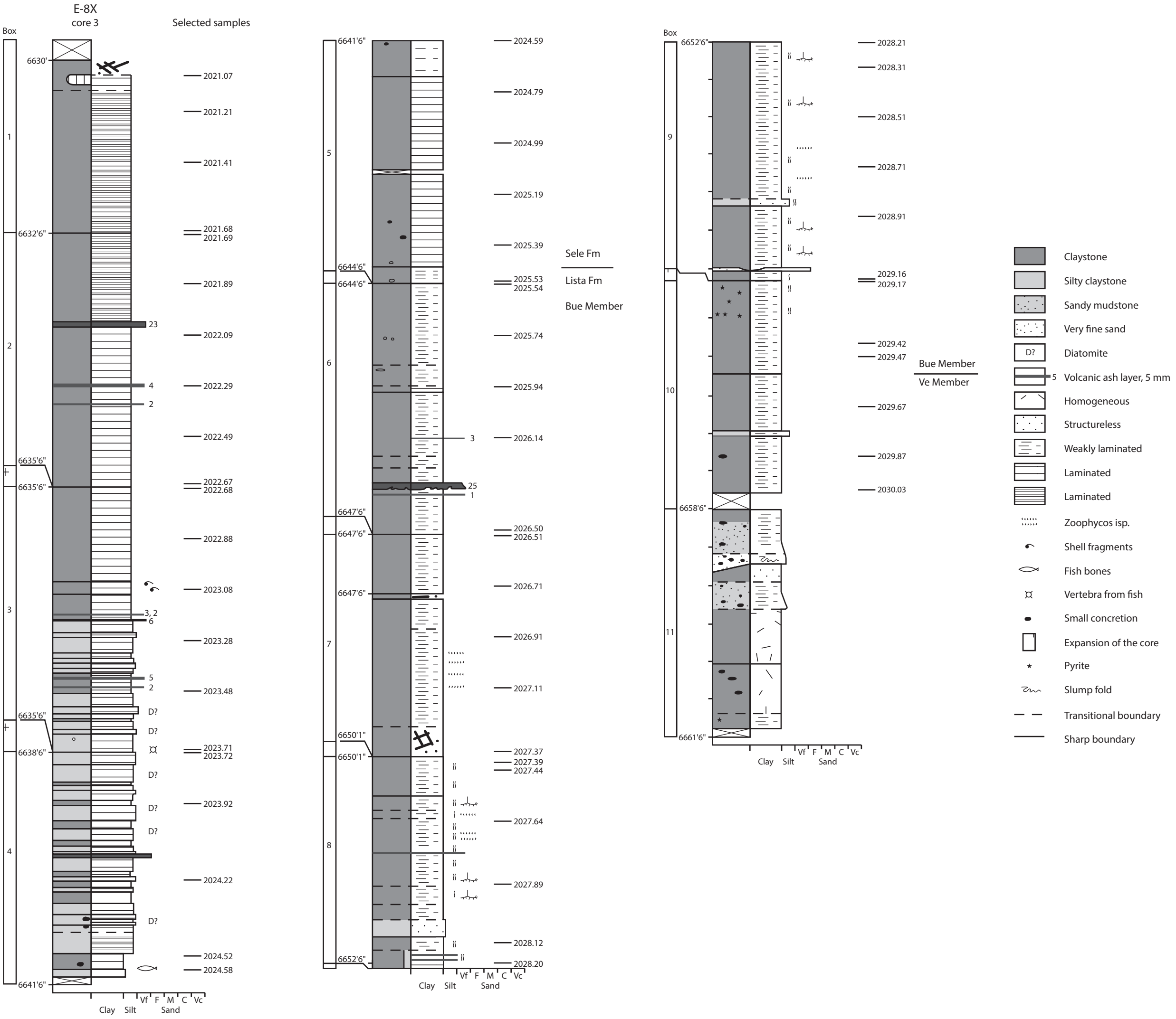

Supplementary Fig. 2. Sedimentological log of the studied interval of E-8X (Core 3, Boxes 1–11). Sediments assigned to the Lista Formation (uppermost Ve Member, and Bue Member) and to the Sele Formation (lower part). The lower boundary of the Sele Formation corresponds to the start of the PETM interval (onset of laminations). Although samples were taken approximately every 1-2 cm, only a few of the samples are indicated to the right of the log (in meters). The scale on the left of the log (in feet) is the original core scale before the core expanded.

Supplementary Table 1. Absolute counts of dinoflagellate cysts for core E-8X.

Site	Depth (m)	Box	Box depth (cm)	*Achomosphaera* spp.	acritarch indeterminate (3 long spines)	*Alterbidinium* spp.	*Apectodinium augustum*	*Apectodinium* spp.	*Areoligera* spp.	*Caligodinium aceras*	*Cerodinium depressum*	*Cerodinium* spp.	chorate cysts - indeterminate	*Deflandrea oebisfeldensis*	*Deflandrea phosphoritica*	*Deflandrea* spp.	dinoflagellate cysts - indeterminate	*Glaphyrocysta exuberans*	*Glaphyrocysta pastielsii*	*Glaphyrocysta* spp.	gonyaulacacean dino. cysts - indet.	*Hystrichosphaeridium tubiferum*	*Lanternosphaeridium* sp.	*Lejeunia* sp.	*Lentinia* sp.	*Micrhystridium* spp.	*Palaeocystodinium golzowense*	*Spinidinium* spp.	*Spiniferites* spp.	*Tasmanites*
E-8X	2021.065	1	6	0	3	6	0	0	0	1	1	0	5	3	4	0	7	0	1	1	0	1	0	1	0	1	0	0	5	0
E-8X	2021.39	1	38.5	0	1	3	0	0	0	0	8	0	17	2	5	3	4	1	1	1	0	0	0	0	0	1	1	6	0	0
E-8X	2021.705	2	2	0	0	2	0	0	0	0	3	1	7	1	2	8	3	0	0	0	0	0	0	0	0	0	0	0	0	0
E-8X	2022.02	2	33.5	0	0	0	0	0	0	0	2	0	1	0	3	1	2	0	0	0	1	0	0	0	0	1	0	1	2	0
E-8X	2022.185	2	50	0	0	0	0	0	1	7	0	1	5	1	0	0	7	0	0	0	0	0	1	0	0	0	0	0	6	0
E-8X	2022.35	2	66.5	0	0	0	0	0	0	0	3	0	7	1	2	0	6	0	0	0	0	0	0	0	0	0	0	0	3	0
E-8X	2022.99	3	31	0	0	1	0	0	0	0	1	0	12	0	0	0	0	0	0	0	0	0	0	0	0	0	0	0	2	1
E-8X	2023.79	4	7	0	0	0	1	0	0	1	0	0	2	0	0	0	2	0	0	0	0	0	0	0	0	0	0	2	0	0
E-8X	2024.64	5	89	0	0	1	1	1	0	0	0	0	1	0	0	0	1	0	0	0	0	?1	0	1	1	0	0	0	1	0
E-8X	2025.21	5	32	0	0	3	1	2	0	0	2	1	4	0	0	0	3	0	0	0	0	0	0	0	0	0	0	0	1	0
E-8X	2025.53	5	0	2	0	1	0	0	0	0	0	0	4	0	0	0	3	0	0	0	0	1	0	0	0	4	0	1	27	0
E-8X	2026.19	6	64	0	0	3	0	0	7	0	0	0	35	0	0	0	19	0	0	0	0	6	0	0	0	21	0	0	53	0
