# Supplementary material for: Paleocene/Eocene carbon feedbacks triggered by volcanic activity
Source: Nat Commun. 2021 Aug 31;12:5186. doi: 10.1038/s41467-021-25536-0 (PMC8408262; doi:10.1038/s41467-021-25536-0)
Supplement: Supplementary file 3 — Description of Additional Supplementary Files [file 41467_2021_25536_MOESM3_ESM.pdf]

Description of Additional Supplementary Files

File Name: Supplementary Data 1
Description: Carbon isotope and total organic carbon data for core E-8X.

File Name: Supplementary Data 2
Description: Mercury data for core E8X and core 22/10a-4.
